# Pharmacological conditioning in the treatment of recent-onset rheumatoid arthritis: a randomized controlled trial study protocol

**DOI:** 10.1186/s13063-019-3777-6

**Published:** 2020-01-06

**Authors:** Meriem Manaï, Henriët van Middendorp, Dieuwke S. Veldhuijzen, Joy A. van der Pol, Tom W. J. Huizinga, Andrea W. M. Evers

**Affiliations:** 1 Faculty of Social and Behavioural Sciences, Institute of Psychology, Health, Medical and Neuropsychology Unit, Leiden University, P.O. Box 9555, 2300 RB Leiden, The Netherlands; 2 Leiden Institute for Brain and Cognition, Leiden University, Leiden, the Netherlands; 3 Department of Rheumatology, Leiden University Medical Center, Leiden, the Netherlands; 4 Department of Psychiatry, Leiden University Medical Center, Leiden, the Netherlands

**Keywords:** Instrumental learning, Conditioning, Pharmacological conditioning, Placebo, Rheumatoid arthritis, Psychological treatment, Randomized controlled trial, Study design

## Abstract

**Background:**

In pharmacological conditioning associations are formed between the effects of medication and contextual factors related to the medication. Pharmacological conditioning with placebo medication can result in comparable treatment effects and reduced side effects compared to regular treatment in various clinical populations, and may be applied to achieve enhanced drug effects. In the current study protocol, pharmacological conditioning is applied to achieve enhanced treatment effects in patients with recent-onset rheumatoid arthritis (RA). The results from this study broaden the knowledge on the potential of pharmacological conditioning and provide a potential innovative treatment option to optimize long-term pharmacological treatment effectiveness for patients with inflammatory conditions, such as recent-onset RA.

**Methods:**

A multicenter, randomized controlled clinical trial is conducted in patients with recent-onset RA. Participants start on standardized pharmacological treatment for 16 weeks, which consists of methotrexate (MTX) 15 mg/week and a tapered schedule of prednisone 60 mg or 30 mg. After 4 months, participants in clinical remission (based on the rheumatologist’s opinion and a targeted score below 1.6 on a 44-joint disease activity score (DAS44)) are randomized to 1 of 2 groups: (1) the control group (C), which continues with a standardized treatment schedule of MTX 15 mg/week or (2) the pharmacological conditioning group (PC), which receives an MTX treatment schedule in alternating high and low dosages. In the case of persistent clinical remission after 8 months, treatment is tapered and discontinued linearly in the C group and variably in the PC group. Both groups receive the same cumulative amount of MTX during each period. Logistic regression analysis is used to compare the proportion of participants with drug-free clinical remission after 12 months between the C group and the PC group. Secondary outcome measures include clinical functioning, laboratory assessments, and self-reported measures after each 4-month period up to 18 months after study start.

**Discussion:**

The results from this study broaden the knowledge on the potential of pharmacological conditioning and provide a potential innovative treatment option to optimize long-term pharmacological treatment effectiveness in patients with inflammatory conditions, such as recent-onset RA.

**Trial registration:**

Netherlands Trial Register, NL5652. Registered on 3 March 2016.

## Background

A promising way to enhance pharmacological treatment effects includes the application of learning mechanisms [1–3]. An example is pharmacological conditioning, which is a form of both classical conditioning, whereby associations are formed between the medication effect and contextual factors related to the medication, and instrumental learning, whereby conscious expectations of the (positive) drug effects will lead to symptom reduction [1, 3–7]. Here, the effect of the active drug in the body is the unconditioned stimulus (UCS) and contextual factors including the look, feel, taste, or scent of the medication, but also the time of day of medication intake or the geographical location, can be the conditioned stimuli (CS). The repeated coupling of the contextual factors with the intake of an active drug, which is accompanied by an unconditioned response (the drug effect), leads to a learned response, the conditioned response (CR), which is similar to the unconditioned response. After forming these associations, the CS in itself (e.g., a placebo tablet looking similar to the active medication) can elicit the CR. A number of studies have shown that pharmacological conditioning can improve treatment effects in different clinical conditions [3, 8–14]. For example, after pharmacological conditioning, comparable treatment effects and reduced side effects have been shown after the administration of reduced or subclinical dosages of active medication combined with placebo medication in comparison to regular active medication dosages [8–13]. The formation of associations between contextual factors and the medicinal effect is a key factor in this process and occurs mainly automatically after the active medication had its effect on disease symptoms [3, 15, 16].

Fast and strong learning effects of pharmacological conditioning with contextual factors have been shown in continuous reinforcement schedules, wherein active medication is provided on every occasion of medication intake [17–19]. In partial or intermittent reinforcement, wherein active medication is provided on only some occasions, satiation effects, whereby familiarity with the association between contextual factors of the active medication and its pharmacological effects in the body reduces responsiveness, are significantly smaller than in continuous reinforcement schedules. In addition, such schedules are also more resistant to extinction of pharmacological conditioning effects [17–19], probably due to the lack of consistent reinforcement during the learning phase, which makes it difficult to recognize the transition to the extinction phase [1, 17–19]. Based on this evidence, an initial continuous reinforcement pharmacological schedule followed by a partial reinforcement schedule is considered to be most optimal for pharmacological conditioning [3, 8]. With an initial continuous reinforcement schedule, strong associations between contextual factors of the active medication and its pharmacological effects are established fast, while following up with a partial or intermittent reinforcement schedule, allows the maintenance and extension of these strong associations over a longer period of time [8]. Evidence for the potential clinical value of pharmacological conditioning by using continuous and partial reinforcement schedules is provided in a study by Ader and colleagues [8], where patients with psoriasis were conditioned to either continuous or partial reinforcement schedules or a combination of these schedules. The strongest learning effect was found for the combination of an initial continuous reinforcement schedule, followed by a partial reinforcement schedule. This combined reinforcement schedule was associated with significant treatment effects on a reduced dosage of active medication comparable to the treatment effects of the full dosage [8]. Although promising results arose from this and other studies, as of yet, pharmacological conditioning has only been studied in a few clinical populations, including patients with psoriasis, irritable bowel syndrome, Parkinson’s disease, attention deficit hyperactivity disorder, and multiple sclerosis [8–13]. In order to broaden the knowledge on the potential of pharmacological conditioning and generalize the effects to other clinical populations, we designed a double-blind, randomized clinical trial to compare standard treatment to treatment with pharmacological conditioning in a population of patients diagnosed with recent-onset rheumatoid arthritis (RA).

RA is a chronic inflammatory autoimmune disease and is characterized by painful and swollen joints, which could lead to radiological joint damage, severe disability, and premature mortality [20–22]. First-line treatment of RA is the use of disease-modifying antirheumatic drugs (DMARDs). Methotrexate (MTX) is the first-choice DMARD in the treatment of RA, as it can slow radiographic evidence of disease progression [21, 23]. The effects of MTX are more robust when combined with either prednisone or a biological agent [24]. However, biological agents come with high economic costs, and, due to considerable side effects of both MTX and biological agents, treatment adherence is suboptimal, especially if medication is taken for a long period of time [25–28]. The most common side effects of MTX are gastrointestinal complaints such as weight loss, nausea, vomiting, stomatitis, diarrhea, and asymptotic elevation of liver enzymes [29–34]. Recent advances in the field of RA treatment with DMARDs have indicated that short and intensive treatment early in the disease can suppress disease activity, improve physical functioning, prevent progression of joint damage, and even result in (drug-free) clinical remission. However, a relatively high percentage (approximately 25–40%) of patients do not reach clinical remission despite this intensive treatment, while even fewer patients obtain drug-free clinical remission (approximately 30%) [21]. Also, such intensive treatment can come with significant side effects and economic costs [21, 27, 35–37]. Pharmacological conditioning may offer a (partial) solution for these issues.

The aim of this study is to assess whether the addition of pharmacological conditioning can optimize the effectiveness of standard pharmacological treatment in a population of patients with early RA. In this study, a pharmacological conditioning schedule is applied, consisting of a continuous pharmacological treatment schedule followed by a partial reinforcement schedule and tapering schedule of an intermittent treatment. It is expected that this pharmacological conditioning leads to a higher percentage of participants in drug-free clinical remission compared to a group that receives standard pharmacological treatment, while both groups receive the same cumulative MTX dosage during the entire study period. Secondary outcome measures include the percentage of participants achieving clinical remission, clinician-assessed and participant-assessed clinical functioning (e.g., disease activity), laboratory assessments (e.g., cytokine levels), and self-report outcomes (e.g., health-related quality of life). This study can offer new therapeutic possibilities in the treatment of diseases that require long-term pharmacological treatment, such as inflammatory conditions in RA.

## Methods/design

**Fig. 1:**
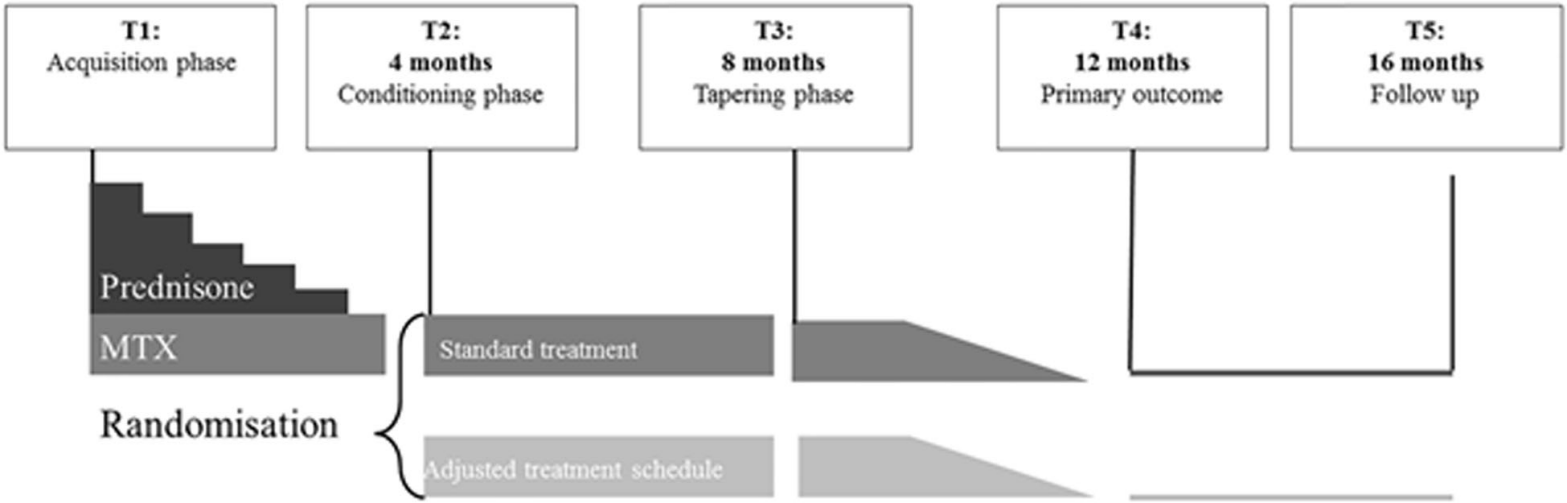
Study design over a period of 16 months with time points 1–5 (T1-T5). MTX: methotrexate

A parallel-group, multicenter, randomized, double-blind, controlled superiority trial is conducted. Figure 1 shows the flowchart of the study design. The Standard Protocol Items: Recommendations for Interventional Trials 2013 (SPIRIT) checklist is presented as Additional file 1.

### Patient involvement

The design of the study, priority of the research question, feasibility, outcome measures, and methods of recruitment were discussed with patients during sessions with a patient panel through the department of Rheumatology at the Leiden University Medical Center (LUMC). Once the trial has been published, participants will be informed of the main results by means of an information letter.

### Data monitoring

In accordance with the Medical Ethical Committee of the LUMC, no data monitoring committee is selected as several independent assessment tools are established to ensure safety and scientific validity and integrity of the current trial. First, following clinical practice routines, all participants are monitored at regular 4-month intervals by their treating clinician. Therefore, suitable and appropriate care for individual participants is monitored and evaluated at regular intervals. In addition, participants are encouraged to contact their treating clinician when disease symptoms worsen or when participants experience medication side effects. Second, the trial design has a set period of time, namely 12 months with a follow-up measurement 4–6 months later, ensuring an adequate amount of time to investigate the intervention effectiveness. Third, an independent data manager at Leiden University assesses various data components at initiation of the trial, every 6 months thereafter, and after the close-out visit. These assessments include, but are not limited to, the inclusion rate and, for the first 3 visits and for 1–10% of following visits selected at random, the presence of informed consents, source data verification (e.g., incidence of clinical remission after 8 months, questionnaires, and blood samples), and serious adverse events. Further, the executive researcher performs random checks to ensure all informed consents are present.

### Study population

Patients with recent-onset RA (according to the American College of Rheumatology (ACR)/European League Against Rheumatism (EULAR) 2010 classification criteria) [20] are recruited from the department of Rheumatology at the LUMC and hospitals in the Medical Delta vicinity in the Netherlands. Eligibility is assessed by the patient’s treating clinician according to specific inclusion and exclusion criteria (Table 1). An eligible patient is asked to sign an informed consent form after receiving written information about the study.

**Table 1:** Inclusion and exclusion criteria

Inclusion criteria	
1	Adult (minimum age of 18 years)
2	Recent-onset RA
3	Fluent in Dutch
4	Able to give informed consent
5.	Clinical remission at month 5 after completing the protocolized pharmacological treatment
Exclusion criteria	
1	Previous therapy with MTX
2	DMARD therapy in the last 6 months or for a period of 3 months or longer, with the exception of anti-malarial drugs
3	Pregnancy or wish to become pregnant during the study, or childbearing potential without adequate contraception
4	Concomitant treatment with another experimental drug
5	Bone marrow hypoplasia
6	Elevated hepatic enzyme levels (ASAT, ALAT > 3 times normal value)
7	Serum creatinine levels > 150 umol/l or estimated creatinine clearance of < 75%
8	Uncontrolled diabetes mellitus (according to the clinician)
9	Uncontrolled hypertension (according to the clinician)
10	Alcohol or drug abuse
11	History of infected joint prosthesis within the previous 3 months
12	Serious infections, such as hepatitis, pneumonia, and pyelonephritis in the previous 3 months
13	Chronic infectious disease, such as chronic renal infection or chronic chest infection with bronchiectasis or sinusitis
14	History of opportunistic infections, such as herpes zoster, within previous 2 months

*MTX* methotrexate, *DMARD* disease-modifying antirheumatic drug, *ASAT* aspartate transaminase, *ALAT* alanine transaminase, *umol* micromole, *NYHA* New York Heart Association

### Procedures

The study is divided into 4 periods of 4 months (total of 16 months, see Fig. 1 for an overview), based on previously studied treatment protocols in patients with RA [21]: period 1 (T1-T2, months 1–4) is the acquisition phase. After initial screening, participants who are eligible for stable standard pharmacological treatment sign the informed consent form and start on methotrexate (MTX) (15 mg weekly) and prednisone (60 or 30 mg daily, tapered to nil in 4 months).

Period 2 (T2-T3, months 5–8) is the conditioning phase. When the treating clinician judges that participants have not attained clinical remission after the first 4 months of standard treatment (based on the 44-joint Disease Activity Score (DAS44), target < 1.6), participants are no longer eligible and are withdrawn from the study. In the case of clinical remission, participants are randomized by the department of Clinical Pharmacology and Toxicology of the LUMC, to one of two parallel groups without further stratification. Participants are randomized by variable block randomization in blocks of six to ensure numerical equality of the treatment groups over time [26, 38–41]. With blocks of six, there are 20 possible ways to equally assign participants to a block. One of these 20 orderings is then randomly selected by means of a random number generator and used to assign participants to one of the two groups according to the specified sequence. The two groups follow different treatment schedules: the control (C) group continues with the standardized treatment dosage of MTX in which the same dosage (15 mg/week) is administered each week. The pharmacological conditioning (PC) group receives an intermittent treatment dosage of MTX in which a high dose of MTX (25 mg/week) is alternated with a low dose (5 mg/week), by interspersing active medication with placebo medication (see “Intervention”). Participants are randomized by the pharmacy at the LUMC. All participants are blind to the pharmacological treatment schedule they receive. Also, all members of the research team who are in direct contact with the participants are blind to the group to which participants are assigned, including the clinicians assessing the primary outcome, the DAS44 at time point 4 (T4: 12 months after baseline), and the researchers who deliver the medication every 4 weeks.

Period 3 (T3-T4, months 9–12) is the tapering phase. Participants who are in clinical remission are tapered off MTX, with dosages either decreasing linearly in the C group or variably in the PC group (see “Intervention”). Participants who are not in clinical remission will discontinue with the study protocol and treatment is continued based on an individualized treatment plan according to their clinician’s best clinical insight and in agreement with the participant. These participants are still followed according to the intention-to-treat principle: if possible and willing, participants do complete all measurements, but discontinue the prescribed treatment regimen of the trial and are further treated at the discretion of their clinician. In order to ensure that any group differences are not due to different dosages of active medication during the study period, both groups receive the same cumulative amount of MTX over the first 3 periods.

Period 4 (T5, months 12–16) is the follow-up phase: as participants are tapered off MTX during the previous period, participants do not receive any medication (or placebo) during this period. The end of study visit takes place at the end of this period.

### Intervention

**Fig. 2:**
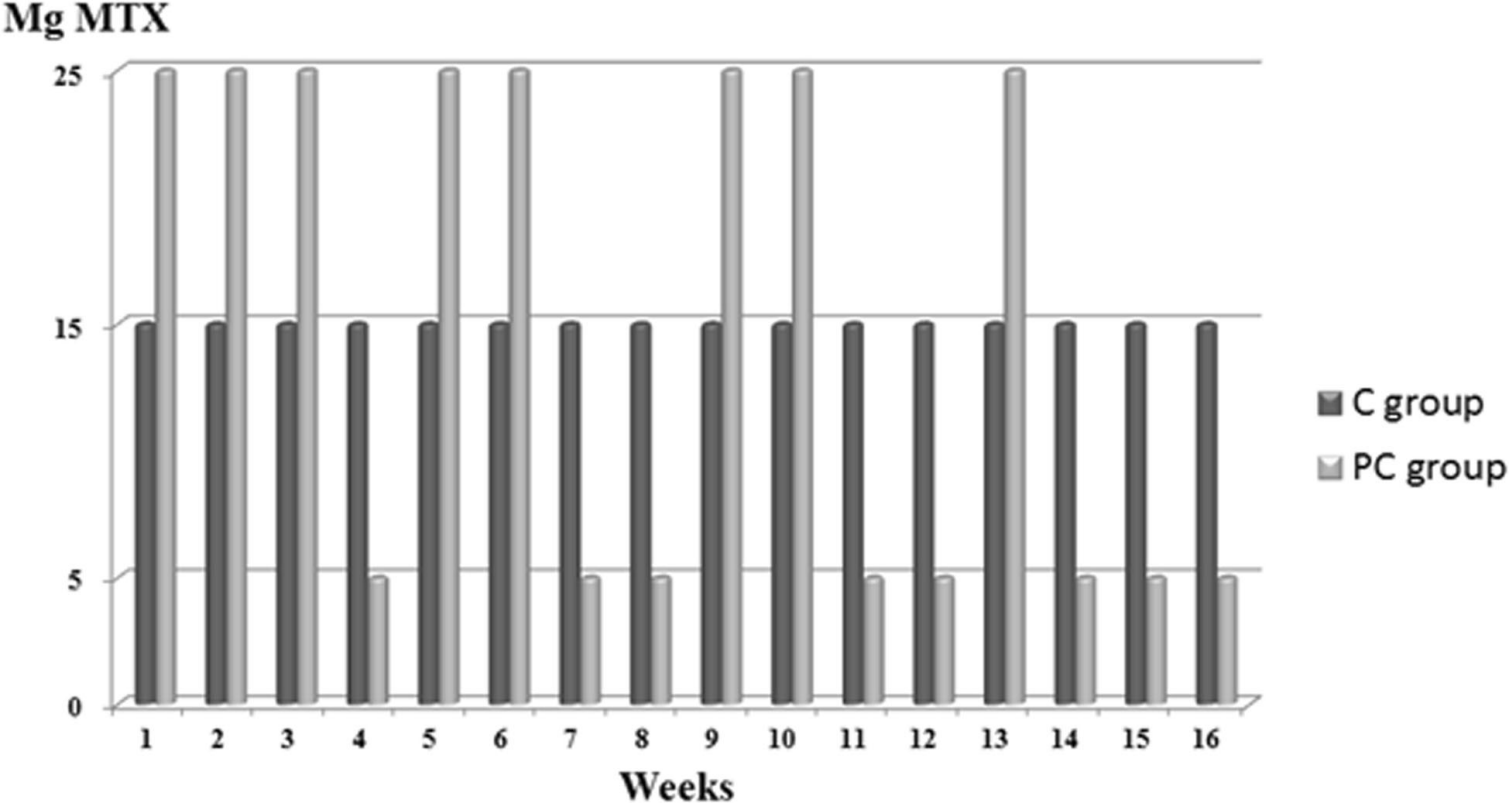
Pharmacological schedule for the conditioning phase. The dosage during this phase is cumulatively the same for both groups. Mg: milligram; MTX: methotrexate; C group: control group; PC group: pharmacological conditioning group

Pharmacological conditioning. A combination of continuous reinforcement in the acquisition phase, followed by partial reinforcement in the conditioning phase, is applied in order to allow for optimal conditioned effects [42–46]. In the acquisition phase, participants are treated with a weekly dose of 15 mg MTX, starting and gradually increasing from 7.5 mg MTX. For the first 2 weeks of the acquisition phase, a total of three tablets of 2.5 mg MTX (7.5 mg MTX) are taken. Starting from week 3 of the acquisition phase, a total of six tablets of 2.5 mg MTX (15 mg MTX) are taken. Medication is taken on the same day, at the same time once a week in order to form associations between contextual factors of the medication intake (CS: e.g., geographical location and time of day of the medication intake, but also the look and feel of the medication itself) and the effect of the active medication in the body (UCS). (see Fig. 2 for an overview). In the conditioning phase, each dose is delivered in a bottle containing 10 identical tablets, with the ratio of active medication versus placebo tablets depending on the MTX dosage, with each MTX tablet containing 2.5 mg MTX. During the conditioning phase, the C group receives a weekly bottle containing a total of 10 tablets with 6 tablets of 2.5 mg MTX and 4 tablets of identical-looking placebos. In the PC group, a variable treatment schedule is applied in order to extend the learned associations between contextual stimuli of the medication and the drug effect in the body over a longer period of time. Participants in the PC group are exposed to weekly high doses of MTX (25 mg; 10 tablets of 2.5 mg MTX), variably interspersed with weekly low doses of MTX (5 mg: 2 tablets of 2.5 mg MTX and 8 tablets with identical-looking placebos). The repeated associations between the CS and the UCS during the acquisition phase will enable contextual factors of the medication intake (CS) to elicit an approximation of the medication effects in the body (UCS) during the conditioning and tapering phases. The end of the conditioning phase is marked by more frequent low dosages in the transition to the tapering phase (see Fig. 2 for an overview).

**Fig. 3:**
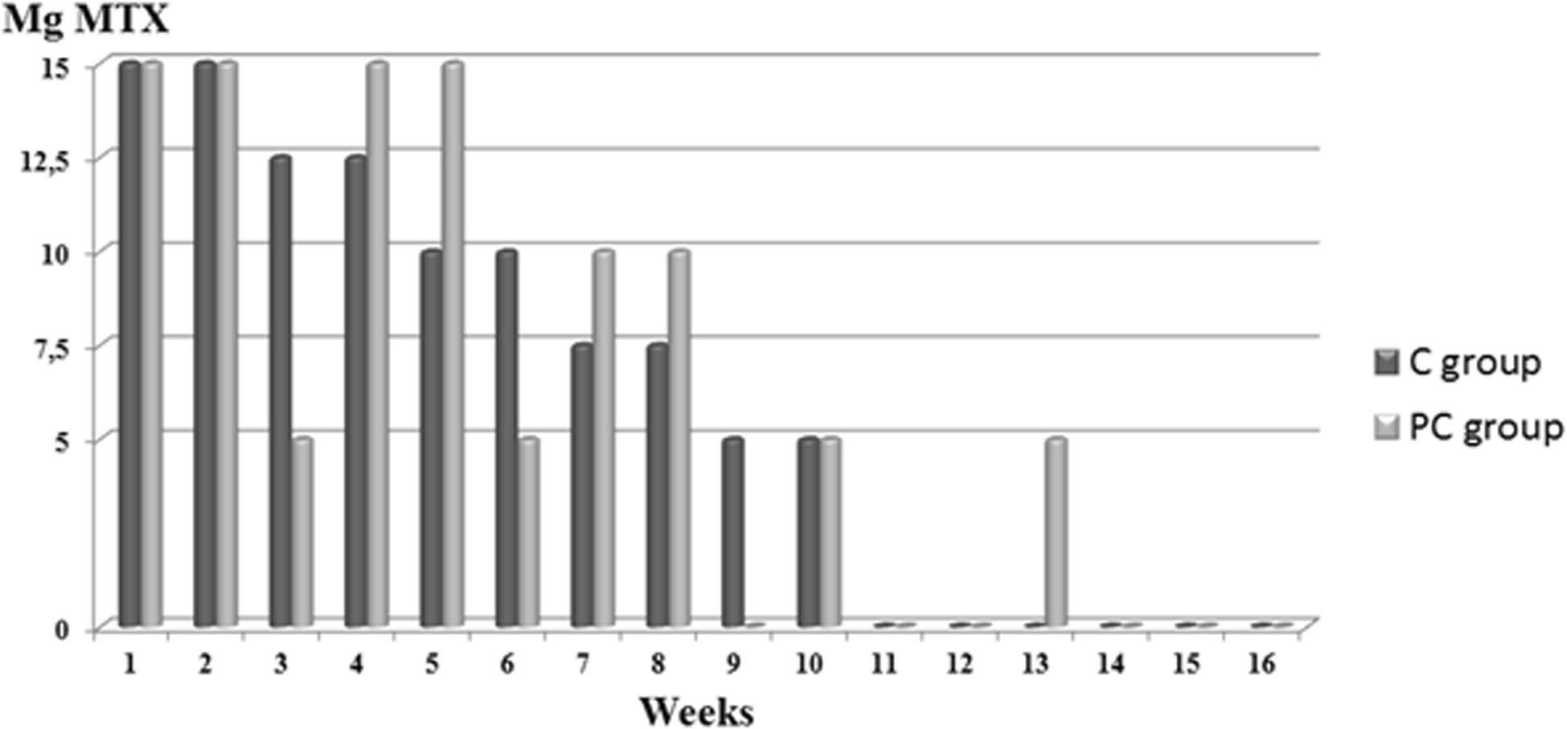
Pharmacological schedule for the tapering phase. The dosage during this phase is cumulatively the same in both groups. Mg: milligram; MTX: methotrexate; C group: control group; PC group: pharmacological conditioning group

During the tapering phase, both groups continue to receive weekly bottles containing 10 identical tablets to be taken on the same day, at the same time once a week, with the ratio of active medication to placebo tablets depending on the MTX dosage, with each MTX tablet containing 2.5 mg MTX. The control group is tapered off linearly in bi-weekly decreases of 2.5 mg MTX (see Fig. 3 for an overview). In order to maintain the learned pharmacotherapeutic responses throughout the tapering period in the PC group, medication is tapered off variably as well, with larger dosages being interchanged with lower dosages instead of a linear decrease in active medication dosage (see Fig. 3 for an overview).

### Concomitant medication

All participants receive a folic acid supplement 5 mg/week to decrease possible MTX side effects. Concomitant therapy with non-steroidal anti-inflammatory drugs (NSAIDs) and with analgesics including paracetamol and tramadol is allowed in both treatment groups. The clinician decides whether a co-intervention is necessary. All participants who need treatment in violation of the study protocol during the acquisition phase (months 1–4) are excluded from the study. Participants who require treatment in violation of the protocol during months 5–16 are handled according to intention-to-treat analysis and no longer receive study medication.

### Assessments

Over a time period of 16 months, all participants visit the hospital every 4 months for 5 assessments in total. At each time point, data are collected by means of a physical examination (swollen and tender joint count), laboratory evaluations (e.g., erythrocyte sedimentation rate (ESR)), and questionnaires (including but not limited to health-related quality of life). The timing of assessments aligns with usual care as much as possible.

### Outcome measures

The primary outcome measure is the difference in the percentage of participants who achieve drug-free clinical remission between the C group and the PC group following the tapering period (T4: 12 months after the start of treatment).

Secondary outcomes include differential effects in the C group and the PC group on clinical measures (e.g., clinical remission at T3-T5), laboratory measures (e.g., ESR), and self-reported measures (e.g., health-related quality of life as measured by the RAND-36 Health Status Inventory [47]) at all assessment points (T1-T5). Also, cost-effectiveness measures are included at T1 to T5. These measures include the Assessment Productivity Cost Questionnaire, which assesses the ability of a person to perform work [48] and the Medical Cost Questionnaire, which assesses medical costs [49].

### Safety monitoring

All adverse events (AEs) are followed until they have abated or until a stable situation has been reached. Serious adverse events (SAEs) are reported within 15 days after the sponsor has first knowledge of the SAE through the web portal ToetsingOnline to the accredited Medical Research Ethics Committee that approved the protocol.

### Statistical analysis

Descriptive statistics of relevant variables are calculated and reported for all data. These include gender, age, and baseline disease activity scores. For the primary analysis examining whether the pharmacologically conditioned (PC) group is superior to the control group (C) in the percentage of participants on drug-free clinical remission, a superiority test is performed by means of logistic regression analysis with drug-free clinical remission (dichotomous variable, yes/no) as the dependent variable and group (C group and PC group) as the between-subjects variable. The difference in proportions allows for direct comparisons with the Induction therapy with MTX and Prednisone in Rheumatoid Arthritis Or Very Early arthritic Disease (IMPROVED) study (ISRCTN registration number 11916566 and EudraCT number 2006–006186-16), which has shown a 32% drug-free clinical remission rate in response to a pharmacological treatment, similar to the C group in the current trial, which was shown to be the most effective pharmacological treatment strategy in inducing remission in early (rheumatoid) arthritis [21]. Thus, the current study can show whether adding the pharmacological conditioning intervention to standard pharmacological treatment is superior to effective standard pharmacological treatment alone. We expect that adding pharmacological conditioning to standard pharmacological treatment will result in a higher percentage of patients in drug-free remission, which is considered to be a relevant clinical outcome. In the case of outliers, as defined by a standardized z-score ≥ 3, sensitivity is analyzed in the sample, excluding the outliers.

In the case of missing data, longitudinal multilevel analysis is performed whereby missing data are handled by means of maximum likelihood estimation. Hereby, missing data are assumed to be missing at random or missing completely at random. Further, non-inferiority testing is performed using two analysis sets; the intention-to-treat (ITT) set, whereby all randomized participants are included in the analyses, regardless of whether or not the protocol was followed, and the per-protocol analysis set, which excludes participants who deviate from the protocol. Tests are performed one-sided with an alpha level of *p* ≤ 0.05, to determine statistical significance of the results. Finally, results are reported in accordance with the Consolidated Standards of Reporting Trials (CONSORT) 2010 guidelines.

### Sample size

Sample size calculations were based on the findings of a study examining pharmacotherapeutic effects in patients with psoriasis, which applied a conditioning design that is most similar to the proposed design of the current study [8]. In this study, for the comparison between the control group (which is similar to our C group) and the partial reinforcement group (which is similar to our PC group), the conditioned pharmacotherapeutic effect had a Cohen’s *d* effect size of 0.61, which is equal to an odds ratio of 3.5 in logistic regression analysis. An a priori power calculation based on logistic regression analysis on the percentage of participants who have achieved drug-free clinical remission (yes/no) at month 12 with two independent groups (Fisher’s exact test) and based on the previous proportion of drug-free clinical remission in the IMPROVED study (32%), a total sample size of 94 participants with RA (47 per group). Based on this effect size, and taking into account a potential 25% drop-out rate, a total sample size of 94 participants with RA (47 per group) needs to be included at the randomization stage (T2) in order to achieve power of β = 0.80 at a significance level of α = 0.05 at month 12 (calculated using Stata Statistical Software: Release 13 and Power Analysis and Sample Size System (PASS) 11, NCSS). In order to include 94 participants at month 5, we need to include 188 participants at month 1: based on a previous study [21] in which 61% of participants achieved clinical remission at month 5 (participants received an average of 25 mg MTX and prednisone during the baseline period), we expect that 50% of participants will be able to complete the baseline period and achieve clinical remission at month 5, as participants in this study will receive an average of 15 mg MTX and prednisone.

## Discussion

Pharmacological conditioning is increasingly accepted as a possible promising therapeutic strategy to optimize treatment outcome, as clinical studies indicate its effectiveness in various clinical populations [8–13]. However, the knowledge of pharmacological conditioning is still limited due to several difficulties. More specifically, so far, patient groups under investigation have differed in their phenotype, making it challenging to assess the value of an intervention in different disease courses and understand their underlying mechanisms. Further, trial outcome measures have mostly used single measure scores to evaluate the efficacy of an intervention [8–13]. However, in many disorders it is difficult to measure disease progression with a single score as symptoms may not only be of a physical nature, but can also include symptoms such as fatigue and quality of life. Therefore, a composite score seems to be more appropriate to measure the efficacy of interventions [50]. The current study aims to overcome these challenges in several ways. First, we set out to define a relatively homogeneous patient population, namely patients with recent-onset RA as defined by the ACR/EULAR 2010 classification criteria who have not received previous MTX therapy or who have had DMARD therapy in the last 6 months or for a period of 3 months or longer, with the exception of anti-malarial drugs. By using this specific sample, potential (negative) associations with previous therapies are prevented from being carried over in the current trial. Second, we have an advanced understanding of the pathogenesis and phenotype of RA [51–54], which might allow for better insight into the interaction between physiological and environmental factors that influence the immune response. Last, our primary outcome measure, clinical remission (yes/no), is based on the DAS44, which is a validated continuous measure that combines multiple constructs into a quantitative index [55–59] and allows meaningful comparisons of the results of the current trial with other clinical trials [59].

Previous studies have focused on achieving similar treatment effects with a lower dosage of active medication or even on solely placebo treatment, as compared to standard treatment [8–13]. In contrast, the aim of the current study is to enhance current pharmacological treatment effects to the same dosage of active medication in a population of patients with recent-onset RA, and to maintain these enhanced effects for a longer period of time as compared to standard treatment by adding pharmacological conditioning to standard treatment. This may not only prevent potential ethical problems of undertreating patients, potentially leading to worse functioning or disease progression if proven insufficiently effective, but also lead to new therapeutic possibilities not only for patients with RA, but also for patients with other chronic diseases requiring long-term (intensive) pharmacological treatment [28].

For the first time, the possibility of pharmacological conditioning is investigated in patients with RA. If this proof-of-concept study shows that pharmacological conditioning of MTX is effective, it could not only induce increased treatment effects of a treatment dosage similar to standard treatment in patients with RA, but the conditioning concept could also be extended to investigate the possibility of achieving (drug-free) clinical remission with a lower dosage, as has been done in other clinical populations [8–13]. A reduction in medication without the loss of its efficacy may lead to fewer side effects and, resultantly, increased medication adherence [23]. Better treatment adherence is directly related to treatment effectiveness and may therefore result in reduced physical, financial, and societal costs [3, 8, 60–62]. Since medication adherence rate estimates in rheumatoid arthritis vary between 30 and 80%, for example due to side effects of the medication [23, 61, 63], it is of importance to find new ways to increase these adherence rates.

In conclusion, in the present study design, an innovative strategy of pharmacological conditioning is proposed that can be used in addition to pharmacological treatment strategies and can optimize the treatment of chronic (inflammatory) disease. This strategy may be employed in order to increase the treatment effects with a comparable cumulative drug dosage over time and achieve earlier drug-free clinical remission and/or longer conservation of drug-free clinical remission, reduce side effects, improve treatment adherence, reduce financial and societal costs, and may provide proof of principle for other clinical populations.

## Supplementary information

**Additional file 1.** SPIRIT 2013 Checklist: Recommended items to address in a clinical trial protocol and related documents.

## Data Availability

Not applicable.

## Abbreviations

ACR: American College of Rheumatology
AE: Adverse events
ALAT: Alanine transaminase
ASAT: Aspartate transaminase
C group: Control group
CR: Conditioned response
CS: Conditioned stimulus
DAS44: 44-Joint Disease Activity Score
DMARDs: Disease-modifying antirheumatic drugs
ERC: European Research Council
ESR: Erythrocyte sedimentation rate
EULAR: European League Against Rheumatism
ICH: International Council for Harmonization
LUMC: Leiden University Medical Center
MREC: Medical Research Ethics Committee
MTX: Methotrexate
NSAIDs: Non-steroidal anti-inflammatory drugs
NYHA: New York Heart Association
PASS: Power Analysis and Sample Size System
PC group: Pharmacological conditioning group
RA: Rheumatoid arthritis
SAEs: Serious adverse events
SPIRIT: Standard Protocol Items: Recommendations for Interventional Trials 2013
UCS: Unconditioned stimulus
Umol: Micromole

## References

[1] Ader R (1993). Conditioned responses in pharmacotherapy research. Psychol Med.

[2] Colloca L, Miller FG (2011). How placebo responses are formed: a learning perspective. Philos Trans R Soc Lond B Biol Sci.

[3] Doering BK, Rief W (2012). Utilizing placebo mechanisms for dose reduction in pharmacotherapy. Trends Pharmacol Sci.

[4] Flaten MA (2009). Drug effects: Agonistic and antagonistic processes. Scand J Psychol.

[5] Kirsch I (1985). Response expectancy as a determinant of experience and behavior. Am Psychol.

[6] Pacheco-López G, Engler H, Niemi M-B, Schedlowski M (2006). Expectations and associations that heal: immunomodulatory placebo effects and its neurobiology. Brain Behav Immun.

[7] Siegel S (2001). Pavlovian conditioning and drug overdose: when tolerance fails. Addict Res Theory.

[8] Ader R, Mercurio MG, Walton J, James D, Davis M, Ojha V, Kimball AB, Fiorentino D (2010). Conditioned pharmacotherapeutic effects: a preliminary study. Psychosom Med.

[9] Kaptchuk TJ, Friedlander E, Kelley JM, Sanchez MN, Kokkotou E, Singer JP, Kowalczykowski M, Miller FG, Kirsch I, Lembo AJ (2010). Placebos without deception: a randomized controlled trial in irritable bowel syndrome. PLoS One.

[10] Sandler AD, Glesne CE, Bodfish JW (2010). Conditioned placebo dose reduction: a new treatment in attention-deficit hyperactivity disorder?. J Dev Behav Pediatr.

[11] Giang DW, Goodman AD, Schiffer RB, Mattson DH, Petrie M, Cohen N, Ader R (1996). Conditioning of cyclophosphamide-induced leukopenia in humans. J Neuropsychiatry Clin Neurosci.

[12] Lidstone SC, Schulzer M, Dinelle K, Mak E, Sossi V, Ruth TJ, de la Fuente-Fernandez R, Phillips AG, Stoessl AJ (2010). Effects of expectation on placebo-induced dopamine release in Parkinson disease. Arch Gen Psychiatry.

[13] Sandler A, Glesne C, Geller G (2008). Children’s and parents’ perspectives on open-label use of placebos in the treatment of ADHD. Child Care Health Dev.

[14] Eikelboom R, Stewart J (1982). Conditioning of drug-induced physiological responses. Psychol Rev.

[15] Benedetti F, Amanzio M, Baldi S, Casadio C, Maggi G (1999). Inducing placebo respiratory depressant responses in humans via opioid receptors. Eur J Neurosci.

[16] Schedlowski M, Enck P, Rief W, Bingel U (2015). Neuro-bio-behavioral mechanisms of placebo and nocebo responses: implications for clinical trials and clinical practice. Pharmacol Rev.

[17] Au Yeung ST, Colagiuri B, Lovibond PF, Colloca L (2014). Partial reinforcement, extinction, and placebo analgesia. Pain.

[18] Ferster CB, Skinner BF (1957). Schedules of reinforcement.

[19] Skinner BF. Contingencies of reinforcement: A theoretical analysis. New York: Appleton-Century-Crofts; 1969.

[20] Aletaha D, Neogi T, Silman AJ, Funovits J, Felson DT, Bingham CO, Birnbaum NS, Burmester GR, Bykerk VP, Cohen MD (2010). 2010 Rheumatoid arthritis classification criteria: an American College of Rheumatology/European League Against Rheumatism collaborative initiative. Arthritis Rheum.

[21] Heimans L, Wevers-de Boer KV, Visser K, Goekoop RJ, van Oosterhout M, Harbers JB, Bijkerk C, Speyer I, de Buck MP, de Sonnaville PB (2014). A two-step treatment strategy trial in patients with early arthritis aimed at achieving remission: the IMPROVED study. Ann Rheum Dis.

[22] Welsing PM, Landewé R, Van Riel PL, Boers M, Van Gestel AM, Van Der Linden S, Swinkels HL, Van Der Heijde DM (2004). The relationship between disease activity and radiologic progression in patients with rheumatoid arthritis: a longitudinal analysis. Arthritis Rheum.

[23] van den Hoogen FH, Benraad B, Hekster YA, van Lankveld W (2009). Adherence rates and associations with nonadherence in patients with rheumatoid arthritis using disease modifying antirheumatic drugs. J Rheumatol.

[24] Van der Kooij S, de Vries-Bouwstra J, Goekoop-Ruiterman Y, Ewals J, Han K, Hazes J, Kerstens P, Peeters A, van Zeben D, Breedveld F (2009). Patient-reported outcomes in a randomized trial comparing four different treatment strategies in recent-onset rheumatoid arthritis. Arthritis Care Res.

[25] Scott DL, Wolfe F, Huizinga TW (2010). Rheumatoid arthritis. Lancet.

[26] Boers M, Verhoeven AC, Markusse HM, van de Laar MA, Westhovens R, van Denderen JC, van Zeben D, Dijkmans BA, Peeters AJ, Jacobs P (1997). Randomised comparison of combined step-down prednisolone, methotrexate and sulphasalazine with sulphasalazine alone in early rheumatoid arthritis. Lancet.

[27] Goekoop-Ruiterman YP, de Vries-Bouwstra JK, Allaart CF, van Zeben D, Kerstens PJ, Hazes JM, Zwinderman AH, Ronday HK, Han KH, Westedt ML (2008). Clinical and radiographic outcomes of four different treatment strategies in patients with early rheumatoid arthritis (the BeSt study): a randomized, controlled trial. Arthritis Rheum.

[28] Wolfe F, Michaud K (2007). Resistance of rheumatoid arthritis patients to changing therapy: discordance between disease activity and patients’ treatment choices. Arthritis Rheum.

[29] Cronstein BN, Bertino JR (2000). Methotrexate: Springer Science & Business Media.

[30] Jeurissen ME, Boerbooms T, Agnes M, Van De Putte L, Doesburg WH, Mulder J, Rasker JJ, Kruijsen MW, Haverman JF, Van Beusekom HJ (1991). Methotrexate versus azathioprine in the treatment of rheumatoid arthritis. A forty-eight–week randomized, double-blind trial. Arthritis Rheum.

[31] Rau R, Herborn G (2004). Benefit and risk of methotrexate treatment in rheumatoid arthritis. Clin Exp Rheumatol.

[32] Suarez-Almazor M, Fitzgerald A, Grace M, Russell A (1987). A randomized controlled trial of parenteral methotrexate compared with sodium aurothiomalate (Myochrysine) in the treatment of rheumatoid arthritis. J Rheumatol.

[33] Weinblatt ME, Polisson R, Blotner SD, Leland Sosman J, Aliabadi P, Baker N, Weissman BN (1993). The effects of drug therapy on radiographic progression of rheumatoid arthritis. Results of a 36-week randomized trial comparing methotrexate and auranofin. Arthritis Rheum.

[34] Scheinfeld N (2004). A comprehensive review and evaluation of the side effects of the tumor necrosis factor alpha blockers etanercept, infliximab and adalimumab. J Dermatol Treat.

[35] Combe B, Landewé R, Lukas C, Bolosiu HD, Breedveld F, Dougados M, Emery P, Ferraccioli G, Hazes J, Klareskog L (2007). EULAR recommendations for the management of early arthritis: report of a task force of the European Standing Committee for International Clinical Studies Including Therapeutics (ESCISIT). Ann Rheum Dis.

[36] Escalas C, Dalichampt M, Combe B, Fautrel B, Guillemin F, Durieux P, Dougados M, Ravaud P (2012). Effect of adherence to European treatment recommendations on early arthritis outcome: data from the ESPOIR cohort. Ann Rheum Dis.

[37] Saunders S, Capell H, Stirling A, Vallance R, Kincaid W, McMahon A, Porter D (2008). Triple therapy in early active rheumatoid arthritis: a randomized, single-blind, controlled trial comparing step-up and parallel treatment strategies. Arthritis Rheum.

[38] Calguneri M, Pay S, Caliskaner Z, Apras S, Kiraz S, Ertenli I, Cobankara V (1999). Combination therapy versus monotherapy for the treatment of patients with rheumatoid arthritis. Clin Exp Rheumatol.

[39] Gerards AH, Landewe RB, Prins AP, Bruyn GA, Goei The HS, Laan RF, Dijkmans BA (2003). Cyclosporin A monotherapy versus cyclosporin A and methotrexate combination therapy in patients with early rheumatoid arthritis: a double blind randomised placebo controlled trial. Ann Rheum Dis.

[40] Landewe RB, Boers M, Verhoeven AC, Westhovens R, van de Laar MA, Markusse HM, van Denderen JC, Westedt ML, Peeters AJ, Dijkmans BA (2002). COBRA combination therapy in patients with early rheumatoid arthritis: long-term structural benefits of a brief intervention. Arthritis Rheum.

[41] Mottonen T, Hannonen P, Leirisalo-Repo M, Nissila M, Kautiainen H, Korpela M, Laasonen L, Julkunen H, Luukkainen R, Vuori K (1999). Comparison of combination therapy with single-drug therapy in early rheumatoid arthritis: a randomised trial. FIN-RACo trial group. Lancet.

[42] Benedetti F (2008). Mechanisms of placebo and placebo-related effects across diseases and treatments. Annu Rev Pharmacol Toxicol.

[43] Price DD, Chung SK, Robinson ME. Conditioning, Expectation, and desire for relief in placebo analgesia. Sem Pain Med. 2005;3:15–21.

[44] Price DD, Finniss DG, Benedetti F (2008). A comprehensive review of the placebo effect: recent advances and current thought. Annu Rev Psychol.

[45] Stewart-Williams S, Podd J (2004). The placebo effect: dissolving the expectancy versus conditioning debate. Psychol Bull.

[46] Thompson WG (2000). Placebos: a review of the placebo response. Am J Gastroenterol.

[47] Vander Zee KI, Sanderman R, Heyink JW, de Haes H (1996). Psychometric qualities of the RAND 36-Item Health Survey 1.0: a multidimensional measure of general health status. Int J Behav Med.

[48] Bouwmans C, Krol M, Severens H, Koopmanschap M, Brouwer W, Hakkaart-van Roijen L (2015). The iMTA productivity cost questionnaire: a standardized instrument for measuring and valuing health-related productivity losses. Value Health.

[49] CB LH-vR, Koopmanschap M, Krol M, Severens H, Brouwer W (2013). Handleiding iMTA medical cost questionnaire (iMCQ).

[50] van Riel P, Renskers L (2016). The Disease Activity Score (DAS) and the Disease Activity Score using 28 joint counts (DAS28) in the management of rheumatoid arthritis. Clin Exp Rheumatol.

[51] McInnes IB, Schett G (2011). The pathogenesis of rheumatoid arthritis. N Engl J Med.

[52] Picerno V, Ferro F, Adinolfi A, Valentini E, Tani C, Alunno A (2015). One year in review: the pathogenesis of rheumatoid arthritis. Clin Exp Rheumatol.

[53] McInnes IB, Schett G (2007). Cytokines in the pathogenesis of rheumatoid arthritis. Nat Rev Immunol.

[54] Upchurch KS, Kay J (2012). Evolution of treatment for rheumatoid arthritis. Rheumatology.

[55] Prevoo M, Van’T Hof MA, Kuper H, Van Leeuwen M, Van De Putte L, Van Riel P (1995). Modified disease activity scores that include twenty-eight-joint counts development and validation in a prospective longitudinal study of patients with rheumatoid arthritis. Arthritis Rheum.

[56] Prevoo ML, van Gestel AM, van T Hof MA, van Rijswijk MH, van de Putte LB, van Riel PL (1996). Remission in a prospective study of patients with rheumatoid arthritis. American Rheumatism Association preliminary remission criteria in relation to the disease activity score. Br J Rheumatol.

[57] Van der Heijde D, Van’t Hof M, Van Riel P, Van Leeuwen M, Van Rijswijk M, Van de Putte L (1992). Validity of single variables and composite indices for measuring disease activity in rheumatoid arthritis. Ann Rheum Dis.

[58] Van der Heijde D, van't Hof MA, Van Riel P, Theunisse L, Lubberts EW, van Leeuwen MA, van Rijswijk MH, Van de Putte L (1990). Judging disease activity in clinical practice in rheumatoid arthritis: first step in the development of a disease activity score. Ann Rheum Dis.

[59] Felson DT, Anderson JJ, Boers M, Bombardier C, Chernoff M, Fried B, Furst D, Goldsmith C, Kieszak S, Lightfoot R (1993). The American College of Rheumatology preliminary core set of disease activity measures for rheumatoid arthritis clinical trials. Arthritis Rheum.

[60] Pugner KM, Scott DI, Holmes JW, HiekeK. The costs of rheumatoid arthritis: An international long-term view. Semin Arthritis Rheum. 2000;29:305–20. 10.1016/s0049-0172(00)80017-7 10805355

[61] Van Den Bemt BJ, Zwikker HE, Van Den Ende CH. Medication adherence in patients with rheumatoid arthritis: a critical appraisal of the existing literature. Expert Rev Clin Immunol. 2012;8(4):337–51. 10.1586/eci.12.23 22607180

[62] Colloca L, Enck P, DeGrazia D (2016). Relieving pain using dose-extending placebos: a scoping review. Pain.

[63] Joplin S, van der Zwan R, Joshua F, Wong PK (2015). Medication adherence in patients with rheumatoid arthritis: the effect of patient education, health literacy, and musculoskeletal ultrasound. Biomed Res Int.

