# Supplementary material for: Pharmacological conditioning in the treatment of recent-onset rheumatoid arthritis: a randomized controlled trial study protocol
Source: Trials. 2020 Jan 6;21:15. doi: 10.1186/s13063-019-3777-6 (PMC6945543; doi:10.1186/s13063-019-3777-6)
Supplement: Supplementary file 1 — Additional file 1. SPIRIT 2013 Checklist: Recommended items to address in a clinical trial protocol and related documents. [file 13063_2019_3777_MOESM1_ESM.docx]

**SPIRIT Figure**

Study Protocol Pharmacological Conditioning RA

|  | **Study period** | | | | | |
| --- | --- | --- | --- | --- | --- | --- |
| **Time point** | **Pre-randomisation** | **T0** | **T1 Month 5** | **T2**  **Months 9** | **T3**  **Month 12** | **T4**  **Month 16-18** |
| **Enrrollment:** |  |  |  |  |  |  |
| Elligibility screening | X |  |  |  |  |  |
| Informed consent |  | X |  |  |  |  |
| Randomization |  |  | X |  |  |  |
| **Assessments:** |  |  |  |  |  |  |
| % Drug-free clinical remission (DAS) |  |  |  |  | X | X |
| Clinical remission (DAS) |  |  |  |  | X | X |
| DAS |  | X | X | X | X | X |
| Cytokines |  | X | X | X | X | X |
| RADAI |  | X | X | X | X | X |
| RAND-36 |  | X | X | X | X | X |
| IRGL |  | X | X | X | X | X |
| CIS |  | X | X | X | X | X |
| MISS |  |  | X | X | X | X |
| BMQ |  | X | X | X | X | X |
| iPCQ |  | X |  | X | X |  |
| iMCQ |  | X |  | X | X |  |
| EQ-5D |  | X |  | X | X |  |
| CQR5 |  |  | X | X | X | X |
| MMAS8-R |  |  | X | X | X | X |
| DNA |  |  | X |  |  |  |
| LOT-R |  |  | X |  |  |  |
| EPQ-R |  |  | X |  |  |  |
| MAQ |  |  | X |  |  |  |
| BIS/BAS |  |  | X |  |  |  |
| Diary |  | X | X | X | X | X |

- RADAI: The Rheumatic Arthritis Disease Activity Index
- IRGL: The Impactof Rheumatic Disease on General Health and Lifestyle
- CIS: Checklist Individual Strength
- MISS: The Methotrexate Intolerance Severity Score
- BMQ: Beliefs about Medicines Questionnaire
- iPCQ: The Institute for Medical Technology Assessment (iMTA) Productivity Cost Questionnaire
- iMCQ: The Institute for Medical Technology Assessment (iMTA) Medical Cost Questionnaire
- EQ-5D: Euroqol-5D
- CQR5: The Compliance Questionnaire for Rheumatology
- MMAS8-R: The Morisky Medication Adherence Scale-Revised
- LOT-R: The Life Orientation Test –Revised
- EPQ-R: The Eynsenck Personality Questionnaire-Revised
- MAQ: The Medication Attitude Questionnaire
- BIS/BAS: The Behavioural Inhibition System and Behavioural Approach System Scales
- Diary: registration of (study) medication intake, experienced complaints, and painkiller use
